# Toward an Ecologically Valid Conceptual Framework for the Use of Artificial Intelligence in Clinical Settings: Need for Systems Thinking, Accountability, Decision-making, Trust, and Patient Safety Considerations in Safeguarding the Technology and Clinicians

**DOI:** 10.2196/35421

**Published:** 2022-06-21

**Authors:** Avishek Choudhury

**Affiliations:** 1 Industrial and Management Systems Engineering Benjamin M Statler College of Engineering and Mineral Resources West Virginia University Morgantown, WV United States

**Keywords:** health care, artificial intelligence, ecological validity, trust in AI, clinical workload, patient safety, AI accountability, reliability

## Abstract

The health care management and the medical practitioner literature lack a descriptive conceptual framework for understanding the dynamic and complex interactions between clinicians and artificial intelligence (AI) systems. As most of the existing literature has been investigating AI’s performance and effectiveness from a statistical (analytical) standpoint, there is a lack of studies ensuring AI’s ecological validity. In this study, we derived a framework that focuses explicitly on the interaction between AI and clinicians. The proposed framework builds upon well-established human factors models such as the technology acceptance model and expectancy theory. The framework can be used to perform quantitative and qualitative analyses (mixed methods) to capture how clinician-AI interactions may vary based on human factors such as expectancy, workload, trust, cognitive variables related to absorptive capacity and bounded rationality, and concerns for patient safety. If leveraged, the proposed framework can help to identify factors influencing clinicians’ intention to use AI and, consequently, improve AI acceptance and address the lack of AI accountability while safeguarding the patients, clinicians, and AI technology. Overall, this paper discusses the concepts, propositions, and assumptions of the multidisciplinary decision-making literature, constituting a sociocognitive approach that extends the theories of distributed cognition and, thus, will account for the ecological validity of AI.

## Introduction

With the growth of multidisciplinary and collaborative health care [1], clinicians have more information and expertise to inform clinical decision-making than ever before [2]. Nevertheless, when confronted with information and knowledge that are (1) not always within the scope of the primary or focal expertise of a clinician and (2) in such quantities that it becomes difficult for the clinician to process reliably and validly and in a timely manner, clinicians can often resort to boundedly rational and, in some cases, incorrect diagnoses, treatment, and other clinical decisions [3]. A response to the interrelated problems of the clinician’s limited absorptive and cognitive capacities has been the integration of artificial intelligence (AI) into health care decision-making [4-6]. However, technological solutions to the problem of limited absorptive and cognitive capacities in multidisciplinary, complex, and collaborative decision-making can introduce new situations [7]. For example, *team science* in clinical settings can come with competing diagnoses and prescriptions for treatment and wellness [8,9]. Furthermore, when new technologies for decision-making are imposed *from above* (eg, by management, rather than organically) or *from below* (eg, at the clinician level, clinicians may not always trust or intend to use those technologies) [10].

The problems regarding trust in AI and the use of AI systems in clinical decision-making illustrate the classic distinction between the rational and descriptive decision-making models. Studies of clinical decision-making demonstrate that the rational model of introducing integrative technologies, including but not limited to AI, into clinical decision-making is not always supported by the data. In other words, rational models of clinical decision-making [6,11,12] and decision-making, in general, are not ecologically valid; they assume perfect information, ideal absorptive and cognitive capacity, optimal trust, and unlimited resources to make a fully and correctly informed decision. The descriptive empirical research demonstrates mixed effects regarding technology-assisted decision-making in clinical settings owing to limited cognitive capacity of the end user (care providers), information overload or lack of data, and suboptimal trust in the technology [7,13,14].

Similar to most technologies, AI can be a boon or bane within the health care ecosystem. With increasing autonomous activities in health care, challenges concerning AI and human factors may manifest evidently at an individual level (eg, awareness and trust), macrolevel (eg, regulation and policies), and technical level (eg, usability and reliability) because many health care AI applications are poorly designed and not evaluated thoroughly [15]. Therefore, human factors and ergonomics (HFE) consideration in health care AI systems has become necessary. If leveraged while developing AI systems, HFE principles and methods can augment its use and adoption without disturbing patient safety or clinical protocols. Of all the possible HFE challenges that AI in health care can cause, suboptimal clinician-AI interaction is significant. Integration of poorly designed AI in health care can complicate the relationships between clinicians and computer (intelligent) systems. Unlike other health care technologies, the complexity of AI is more, as it can interact (through chatbots, automated recommender systems, health apps, etc) with clinicians and patients based on the inputs (feedback) that it receives from them. AI’s output (result generated by the AI) largely depends on the information fed into it—certain types of AI, for instance, reinforcement learning [16], learn and adapt themselves based on user input to optimize the outcome. Therefore, clinician-AI interaction may influence AI performance and, in turn, the clinician’s viewpoint toward it. Optimal and successful clinician-AI interaction depends on several factors, including situation awareness, cognitive workload, working environment, and emotional resources (eg, current state of mind, willingness to use AI, previous experience with AI technology, trust in technology, and others). Most studies on health care AI have ignored (1) ecological validity and (2) human cognition, which may create challenges at the interface with clinicians and the clinical environment. Moreover, there is a lack of sufficient studies focusing on improving the human factors, mainly, (1) how to ensure whether clinicians are implementing it correctly; (2) the cognitive workload it imposes on clinicians working in stressful environments; and (3) its impact on clinicians’ situation awareness, clinical decision-making, and patient safety outcome. Although studies on AI have reported its great performance and potential in medicine [17-19], research breakthroughs (AI performance in research settings) do not necessarily translate into a technology that is ready to be used in a high-risk environment [20], such as health care. In addition, most AI featuring prominent abilities in research and literature are not executable in a clinical environment [21,22]. According to the technology readiness level (TRL), most AI systems, at least in pediatric and neonatal intensive critical care, if not all, do not qualify for implementation [17].

TRL is a gauging system, developed to assess the maturity level of a particular technology [23]. It consists of 9 categories (readiness levels), in which a score of TRL 1 is the lowest and TRL 9 is the highest (Textbox 1).

**Technology readiness levels (TRLs; 1-9).**

**Technologies with TRL 1-4 are executable in a laboratory setting, where the main objective is to conduct research. This stage is the proof of concept.**

- TRL 1: Basic principles of the technology observed
- TRL 2: Technology concept formulated
- TRL 3: Experimental proof of concept developed
- TRL 4: Technology validated in a study laboratory

**Technologies with TRL 5-7 are in the development phase, in which the functional prototype is ready.**

- TRL 5: Technology validated in a relevant environment (controlled setting in a real-life environment)
- TRL 6: Technology demonstrated in a relevant environment
- TRL 7: System prototype demonstrated in an operational environment

**Finally, technologies with TRL 8 and 9 are in the operational phase, in which the primary objective is implementation.**

- TRL 8: System completed and certified for commercial use
- TRL 9: System approved for and implemented in the actual operational environment

For example, in clinical settings, nurses and physicians have demonstrated lack of trust in AI, including machine learning analytics and decision-making tools [7]; numerous other information technologies designed to improve decision-making efficiency and effectiveness, such as medication management systems [13], event reporting systems [14], and electronic health care records systems [24]; and clinical biotechnologies such as gene therapy [25]. There are demonstrations of incorrect use of clinical technologies, such as unwarranted trust and reliance on automated nursing tools, leading to adverse health consequences, including, but not limited to, avoidable fatalities [11] and inappropriate use of medical devices inducing patient harm [12,26]. It is critical to understand that the impact of AI, particularly in health care, is not only a function of the accuracy of its underlying mathematical process but also the cognitive human factors, including trust, perception, usability, and safety. Therefore, to minimize errors caused by health care AI (as noted in other health information technology [HIT] literature, such as electronic medical records), a holistic approach, recognizing health care as a dynamic sociotechnical system in which subelements interact, is necessary.

## Objective

This study aimed to propose a descriptive conceptual framework derived from cognitive human factors and decision-making literature. Note that this framework is not a rational model. Then, future studies can leverage this framework and inform the eventual development of a prescriptive framework for optimal AI-clinician interactions. The proposed framework can be best used for mixed methods studies. In other words, the descriptive conceptual framework will help to capture the interactions between clinicians and AI. The prescriptive framework (guided by experimental study findings) will help to develop better AI-clinician interactions.

The novelty of the descriptive framework presented in this study is that it uses systems thinking and combines multiple descriptive (vs rational) human factors approaches to understand clinician-AI interactions in decision-making. Although human factors considerations in clinical decision-making can augment the intended positive impacts of integrative decision-making technologies such as AI, so far, there are few studies on how and the extent to which clinicians use AI in diagnostic and health care decision-making. In addition, the predominance of empirical studies of AI in clinical settings focuses on the technical aspects of AI-driven diagnostic and care decision-making, that is, the plethora of machine learning algorithms and high-dimensional data that AI entails [27]. The few studies on human factors in the use of AI in decision-making are not focused on clinical samples and contexts, but rather on nonclinical applications in other industries and sectors of the economy, including, but not limited to, military [28]; transportation [29]; and organizational design, in general [30].

## Framework Development

The health care management and medical practitioner literature lack a conceptual framework for capturing the impact of AI from a systems perspective and simultaneously understanding clinician-AI interactions that are ecologically valid, specifically focusing on how such interactions may vary based on human factors such as expectancy, trust, cognitive variables related to absorptive capacity and bounded rationality, and concerns for patient safety. To derive the conceptual framework, this study leverages (1) literature on systems thinking and AI in medical practice, (2) information use in human decision-making, (3) trust and informing decisions with AI, and (4) patient safety and informing decisions with AI.

### Systems Thinking and AI in Medical Practice

#### Overview

Technological advancement and diffusion of innovation are supporting an expeditious transformation in the structures and institutions in veritably every facet of life, and medical practice is no exception. Technologies can now facilitate the accomplishment of activities that humans once considered impossible and are responsible for substantial social and public policy changes in health care. For example, Widmer et al [31] discussed the convergence of health care policy reform in the United States with technological advancements and social shifts as support for the great use of AI in health care practice. They argued that these are transformational forces that influence the capacity to develop complex solutions to problems in medicine. These solutions are in the form of technologies that often rely on AI to support decision-making. Qadri et al [32] surveyed the current landscape of new health care technologies, uncovering the ubiquity of AI and tools dependent on AI in medicine. For example, the impact of the health care Internet of Things on health care information technology has been substantial [32], as the immensity of technological innovation relentlessly pushes forward as systems become increasingly smart and widespread. As these systems become an integral part of health care, systems thinking will become increasingly essential because of the complex nature of the task-technology fit required in health care.

The health care industry has witnessed several design errors in both technologies and clinical workflow. Integrating HITs that are not designed and not tested properly is highly likely to contribute to new categories of technology-induced errors, often new to the health care domain. Such errors usually manifest in the complex interaction between health care providers and HIT during actual clinical use. For example, in the recent past, surgical robots were responsible for 144 patient deaths and 1391 patient injuries [33]. Once integrated, such technologies can also alter the existing clinical workflow. For example, integration of AI into the clinical workflow without considering its impact on clinicians, patients, hospital expenses, workflow speed, insurance claiming process (previous authorization), and other aspects can disrupt the overall care process. For example, given the dependence of AI on data, it is feasible to assume that even the best AI systems will sometimes be wrong, leading to compromised patient safety. Although clinical errors and near misses are common in health care, AI errors can be significantly unique. First, errors arising from AI systems can become widespread without being identified by clinicians, causing system-wide error—rather than the limited number of patients injured by any provider’s error. Second, tracking AI errors can also become highly challenging, mainly when powered by deep learning algorithms. Such a complex system (AI) can make root cause analysis very daunting and almost impossible owing to its inherent opaque nature. The performance of AI systems largely depends on the data on which they have been trained. As the existing data repositories are biased, AI integration without addressing issues regarding data quality can escalate health care biases.

Health care authorities must account for several extrinsic factors such as clinicians’ willingness to use AI in their clinical practice, access to the duration and frequency of AI training required by clinicians with different expertise, and feasibility of personalizing AI for individual clinicians and patients. In addition, doctors and nurses, the potential users of AI in a hospital, can also misuse the system either owing to lack of AI literacy or poor usability of AI. Therefore, a systems thinking approach is essential for the safe integration of AI.

In addition, AI-based technologies may not work well for patients with rare diseases, as their data are not adequately available. Health care authorities will also have to ensure that, over time, clinical experts do not become deskilled or permanently replaced owing to AI implementation. In other words, safe and sustainable integration of AI requires a systems approach in which all interactions between different health care stakeholders are considered.

**Figure 1:**
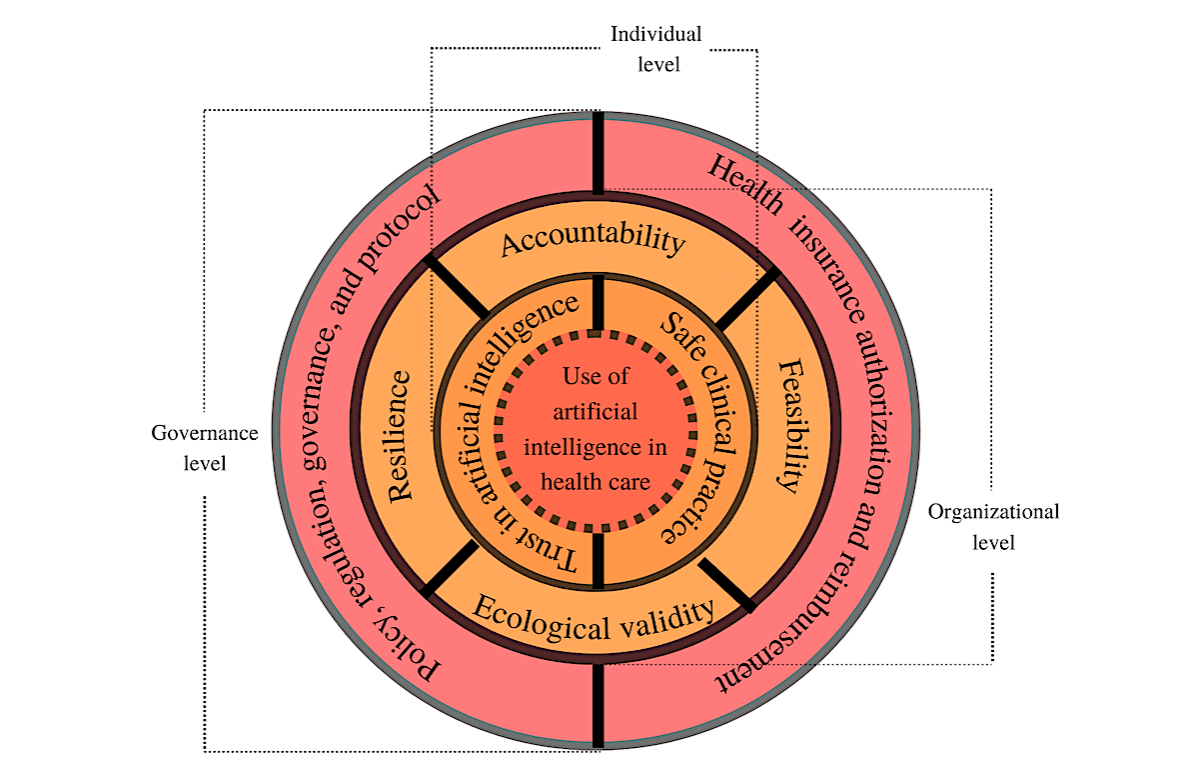
Factors influencing the use of artificial intelligence in health care—a systems viewpoint.

Similar to any complex system, subsystems of health care and AI can be shaped by several factors at three major levels: (1) governance—policies, regulations, and protocols; (2) organizational [34]—accountabilities, resilience, ecological validity, and feasibility; and (3) individual—trust in AI and safe practices (Figure 1).

#### Governance Level

In this study, AI governance has been defined as a group of systems that regulate and control AI within the large health care ecosystem. It steers organizational objectives and risk monitoring to achieve optimized performance. In other words, AI governance is a *system of systems* that requires a holistic approach, incorporating strategic planning at all organizational levels. Existing studies have confined health care AI governance within the boundaries of organizational structure and processes for clinical decision-making, transparency without exploiting proprietary rights, fairness of the technology, and accountability [35].

Nevertheless, many critical factors have not been considered. Resilience; ecological validity; protocols for safe practices using AI; engagement; and responsibilities of stakeholders, including insurance providers; and human factors should also be included as significant components of health care AI governance. *Systems thinking* in health care can help regulatory authorities and organizations to perceive the integration of AI and health care as a merger between 2 complex systems. In other words, a systems approach will allow us to capture and understand how the dynamic relationships between various factors, such as policies and protocols, impact the resilience and feasibility of the incorporation of AI into the health care ecosystem. Clearly defined policies and protocols and involvement of all stakeholders will also help to resolve the current concerns regarding AI accountability—who should be responsible for a flawed AI system or incorrect AI output? From a human factors perspective, the systems approach can advocate for the ecological validity of AI, ensuring that the technology is appropriately designed and developed for a given uncontrolled environment. Addressing these concerns can increase the likelihood of AI acceptance among clinicians, by augmenting their initial trust in the technology.

#### Organizational Level

Further expanding on the subcomponents of Figure 1, AI governance in health care should account for (1) resilience thinking approach, (2) accountability, and (3) ecological validity of AI. *Resilience thinking* is a holistic way of investigating how interacting systems of clinicians and clinical environment, including AI technologies, can be best managed during uncertainty or systemic errors.

This study defines *AI accountability* as a process in which health care practitioners have potential responsibilities to justify their *clinical actions* to patients (or families) and are held liable for any impending positive or negative impact on patient health. While using an AI-based decision support system, only clinicians are held accountable if they decide to follow AI, resulting in patient harm. Clinicians are also held responsible if they deviate from the standard protocols [36]. This may be worrisome because, under such circumstances, clinicians will only follow AI if it matches their judgment and aligns with the standard protocol—making the AI underused. According to our recent survey (*institutional review board 2022-007* approved by the Stevens Institute of Technology, the United States) consisting of 265 clinicians actively practicing in the United States, lack of AI accountability is a significant hindrance to AI adoption in health care. Clinicians hesitate and refuse to use AI as they do not want to take responsibility for faulty AI. Participants in our survey advocated for contractual agreements with patients and policies to safeguard them from AI errors and related patient safety issues. Textbox 2 shows some of the responses provided by clinicians.

**Solutions provided by health care practitioners to address the lack of artificial intelligence (AI) accountability—categories and sample responses.**

**Contractual agreement with patients**

- “I think a solution would need to be having patients sign informed consent for AI to be used in their care and that decisions made by the AI cannot reflect on the provider’s care.”
- “AI should only be used if a patient is willing to fill out a questionnaire regarding the pros and cons of using AI and the potential harm or good, releasing the practitioner along with the potential outcome they may or may not achieve.”
- “[I] think the patient should sign a waiver if AI is used.”

**Policies and safety measures**

- “Use it in conjunction with training and safeguards that are in place now.”
- “The use of AI would need to be regulated. The manufacturers should take full responsibility for any negligent or bad decisions about patient care.”
- “[I] would not want to be held accountable for AI recommendations. Creating policies to protect clinicians would be important.”

Being a complex system, subsystems of health care establishments are shaped by several internal and external factors. This complexity of the health care system can be well explained by using human factors approaches such as the Safety Engineering Initiative for Patient Safety (SEIPS) framework [37]. Developed by Carayon et al [37], the SEIPS framework is partly based on the well-known structure-process-outcome model of health care quality by Donabedian [38]. It is arguably one of the most acknowledged and published systems-based human factors frameworks in health care. SEIPS framework illustrates the dynamic interactions between people (patients and clinicians), technology (AI in this context), tasks (clinical activities to support patient safety and health), and environment (clinical and organizational setting). However, no studies have used the SEIPS framework to understand the impact of AI on health care from a systems perspective.

#### Individual Level

Diligent scrutiny is essential for medical practitioners when considering the application of new technologies in patient care. There are limitations to the benefits of AI in medical practice. Failing to acknowledge them when engaging in innovative decision-making, especially when human lives are at risk, can result in system accidents. Research [39] has discussed the limitations attached to AI application in medicine, focusing on its application in oncology. They noted that machine learning plays a substantial role in oncological practice. Machine learning, which is a subset of AI, involves computers’ ability to learn autonomously through data input [39]. In oncology, benefits of machine learning include application in risk modeling, engaging in diagnostic and staging investigation, prognosis prediction, and therapy response prediction. Limitations persist when using AI, such as costs, overdependence on data quality, black box effect, and obtaining trust in and acceptance of machine learning technology [39]. Mendelson [40] echoed some of the limitations discussed by Khan et al [39], noting that physicians cannot rely on AI alone when making decisions about the findings from breast imaging examinations. Mendelson [40] described the preferred role of AI as being supportive of diagnosis and patient management.

Although physicians and researchers describe several limitations to accepting AI owing to its analytical abilities and biases, other human factors have often been neglected. Although the refinement of methods and procedures used in AI for decision-making continues to advance, further exploration of leveraging human factors principles is mandatory. The solution to safeguard AI and patients is in the acceptance of systems thinking approach to medical care, in which physicians incorporate AI in a role that is, as Mendelson [40] noted, supportive in nature. As described by Khan et al [39], the problem of trust with AI appears to be well founded because of the black box effect, in which the AI delivers results with a solution; however, the rationale for the solution cannot be described. Hashimoto et al [41] noted that although the black box effect exists, efforts are underway to design solutions that can mitigate it in medical practice. The black box results obtained from neural network methods can lead to the correct response [41]. However, physicians cannot rely on the results from AI alone at this point, when making decisions that have life-or-death implications for patients. The system cannot explain itself. Although physicians can learn more about AI to understand the results better [40], the problem of human trust in AI remains challenging because the design of AI does not support transparency [40]. Hence, *the limitations of AI are not based on AI alone, but they are based on the relationship between AI and its users’ lack of understanding of the technology*. *Not all the trust needed to rely on AI can come from better design features. Some must come from great acceptance of the technology, and the interdisciplinary nature of systems thinking can play a role in improving the relationship between humans and AI in medicine*. However, it is essential to keep in mind the dynamic nature of trust, where the user needs to have a priori trust in technology to use it for the very first time. Moving forward, their trust in it can become a function of their experience with the technology and its effectiveness.

In addition to the possibility of patient harm caused by disruption to health care delivery, the complexity of how systems fit together can result in system accidents. Kappagoda [42] discussed the problem of system accidents in aviation to illustrate the potential for problems when there are design deficiencies, poor maintenance practices, and failures in oversight. Similarly, poor AI design in health care can lead to patient harm, where clinicians can misinterpret AI information or click on the wrong option on the AI display. Inadequate maintenance, that is, not retraining the AI with new patient data, can compromise its prediction accuracy, and thus hinder patient safety [43]. In addition, sometimes, bedside care providers make clinical decisions that do not necessarily fall within the standard guideline (for specific patient types) or skip the prescribed clinical steps (under excessive workload) to accomplish a particular clinical goal promptly [44]. Therefore, AI developers should account for such human behavior while designing their products, so that AI can serve as a support and not as a hurdle in the everyday clinical workflow.

Some assurances related to medical devices exist in the United States. These include the International Organization for Standardization 13485 quality standards for medical devices [45] and 21 Code of Federal Regulations 820.3(l) [46]. Although these certifications and regulations exist to protect patients, medical personnel can still preside over a case in which system accidents harm a patient. Hence, although AI can be substantially beneficial to patients and be a helpful tool for supporting the staff's decisions, medical professionals must engage in systems thinking when assessing care strategies.

### Information Use in Human Decision-making

The motivation to use or not use certain information in decision-making is complex, and several theoretical perspectives, such as Situation Awareness and Expectancy Theory, can support the understanding of this motivation. Soltani and Farhadpour [47] investigated user motivation toward using information services. The framework for their study was the expectancy theory. They found that user motivation to use an information service was significantly influenced by awareness of results value and perceptions of the accessibility [47]. *Although expectancy theory appears to play a role in describing why AI is used or not used, other human factors can support in predicting user behavior*. O’Reilly [48] examined the variations in the use of information sources to understand the impact of quality and accessibility of information as factors influencing its use. O’Reilly [48] found that the frequency of use was the most significant influencer of use.

The association of absorptive capacity with the ability to use information is another essential facet of the psychology of decision-making. Results from the study by Liao et al [49] indicate that absorptive capacity has an impact on innovation, but information use aimed at innovation was found to be complicated [50]. Schmidt [50] discovered that the determinants of absorptive capacity are different, depending on the type of knowledge absorbed. Therefore, absorptive capacity is path-dependent in how it leads to information use. This is a complexity that constrains decision-making. In decision-making research, perceived relevance of and access to information are critical to understanding information use. One of the first studies to understand the effects of information relevance on decision-making in complex environments was by Streufert [51]. The framework for her study was the complexity theory. She noted information relevance as a factor that affected complex decision responses, but the same element (information relevance) failed to influence simple decision responses [51]. These findings are critical to understanding the significance of information in decision-making research because they support the essential nature of *situation awareness* among decision makers. *Her conclusion that complex decision-making is affected by relevance and simple decision-making is affected by information load—is a critical finding*, placing some limitations on the complexity theory. Citroen [52] explored the role of information in strategic decision-making by executives in organizations. The approach requires executives to collect and use information in a structured process that supports the elimination of uncertainty in the decision-making process. The findings of Streufert [51] and Citroen [52] support the role of situation awareness as a factor that influences information use among decision makers.

The acceptance of information in decision-making is another pivotal factor in decision-making research. According to a well-established model called technology acceptance model, acceptance is associated with ease of use and usefulness in decision-making [53]. The inclusion of information in decision-making would appear to be important. However, its inclusion and tools such as decision support systems remain as a challenge for decision makers. Todd and Benbasat [54] examined the use of information in decision-making. Their study was critical of assuming that managers who have more information will make better decisions. They found that *the conservation of effort* occurred when managers were presented with more information. Tools such as decision support systems did not result in a great likelihood of information being used in the decision-making [54]. These findings can have substantial implications for studies on human decisions formed by AI, because AI often involves aggregating several piles of data to construct a comprehensive understanding of the phenomenon under investigation. However, if what Todd and Benbasat [54] proposed is consistent with decision makers’ current approach to information, the aggregation of data to create elegant models to understand a phenomenon will go in vain. Studies exist on why individuals choose to rely on information systems for decision-making. Snead and Harrell [55] examined the decisions by the management to use decision support systems using expectancy theory. The findings indicate that the expectancy force model can determine managerial behavioral intentions to use decision support systems [55]. Behavioral theory is helpful for these studies because it can support the assessment of why people use systems without previous experience with them based on intention and motivation.

Absorptive capacity is associated with the use of AI, and the findings from AI are critical. Absorptive capacity is also essential in decision-making related to innovation and depends on how a user optimizes information system capabilities. Moreover, absorptive capacity also impacts AI in terms of industry innovation. *A limitation of AI use is the lack of user understanding of tools or substantively interpreting findings*. Shi et al [56] discovered that AI use creates challenges in terms of limitations, such as limited knowledge transfer. The extent to which workers are trained to use AI tools and interpret their findings is limited. Therefore, absorptive capacity in business settings where AI is used is limited by workers’ capabilities.

The knowledge and relevance of AI are also essential to consider while supporting decision-making. Prevedello et al [57] examined the challenges of AI use in medical settings. They noted a difference between expectations and AI application in clinical settings where AI’s role in tasks, such as radiology, would expeditiously advance purely from a technical standpoint without addressing all the user needs from a human factors perspective. Prevedello et al [57] noted that AI should be a part of developing clinically relevant outcomes and that AI should play a role in decision-making in the future. However, this is also a prediction that Prevedello et al [57] found to have gone unfulfilled from previous studies. Pomerol [58] discussed the issue of AI and human decision-making. He described AI as sharing several relationships with other types of quantitative analytical procedures in that each is useful in diagnosis. He also noted that a critical limitation of AI was the lack of capacity for look-ahead reasoning, where uncertainty and preferences are crucial factors to consider [58].

*Acceptance of AI in decision-making is a critical technological concept in which the ease of use and usefulness of AI is examined and determined. The use and benefit of AI in decision-making are substantially challenged by lack of knowledge of the technology or its potential capabilities*. Chan and Zary [59] discussed the applications and challenges of AI implementation in medical education. One of the major factors restraining AI use in the medical profession is that the medical school curriculum fails to develop future medical professionals to understand AI algorithms [59]. The lack of knowledge and development results in limited use of the tool. A critical limitation to the use of AI in the future appears to not be caused by the constraints or complexities of the technology, but instead by the decision to use the technology by humans [59]. Sohn and Kwon [60] examined several technology acceptance theories to understand which framework best fits the acceptance of AI. Their study included the technology acceptance model, theory of planned behavior, unified theory of acceptance and use of technology, and value-based adoption model. The findings supported the value-based adoption model as the best model to determine user acceptance of AI. Specifically, the factors found to have the most significant impact were *enjoyment* and *subjective norms* [60]. These findings provide evidence that the motivation to use AI is driven more by interest in technology than the utilitarian aspects of AI.

### Trust and Informing Decisions With AI

Trust in technology is influential in several contexts, including those where computer-mediated communication is used for work team communication [61], supporting customers or clients engaging in electronic channels, e-commerce [62], and aviation activities [63]. Trust in technology delineates from trust in humans in many different ways [64,65], in that trust in humans is associated with interpersonal relationship qualities. In contrast, trust in technology is associated with reliability and performance. Nevertheless, trust remains an important aspect of the human experience in technology [65].

Trust in technology appears to be consistent with theories such as the expectation disconfirmation theory. This theory is related to the satisfaction an individual has with experience related to whether their beliefs were confirmed during an experience and how expectations and perceived performance affected their initial beliefs [66]. Trust in technology is complex for many reasons. A reason for the complexity of trust in technology is that there are risks and uncertainties associated with technology. Li et al [67] examined trust in new technology in the context of the workplace. They found that initial trust formation relies on several factors, including trusting bases, beliefs, attitudes, organization’s subjective norms, and trusting intentions. Other studies involving technology assume that trust in technology can be formed through governance in the organization. Winfield and Jirotka [34] discussed the development of a framework for ethical governance, pertaining to robotics and AI systems in organizations. Factors that were considered included ethics, standards, regulation, responsible research, innovation, and public engagement. These factors were deemed essential in the development of trust between the technology and public. The problem with this approach is that it does not consider human factors such as users’ perception of technology, perception of risk associated with it, and its impact on users’ cognitive workload and situation awareness.

Most relevant to this research is the issue of trust in medical technology. Montague et al [68] examined trust in medical technology and sought to describe medical technology as a distinct construct from trust in general technology. A review of the literature on trust in technology was included in their study. Their literature discussion included the assertion that previous study findings support a lack of difference between trust in humans and trust in technology. However, McKnight et al [64] and Lankton et al [65] have included findings and discussion, which indicate a substantial difference between trust in humans and trust in technology. The difference in the findings supports further investigation. The existence of an entirely separate construct describing or measuring trust in medical technology, aside from trust in general technology, should not be considered in subsequent studies to conclude whether a separate construct exists. However, it is beyond the scope of this study.

Specifically, trust in AI remains as an important issue and will grow significantly with time as AI becomes increasingly infused into the products we use in everyday life. AI continues to create some difficulty among researchers regarding how AI should be trusted. AI uses large amounts of data to support decisions that receive attention and consideration based on strong predictability, while not mimicking humans’ thought processes. Hurlburt [69] discussed AI as a technology that continues to increase its reach and that people have become increasingly dependent on the use of AI in their everyday lives. The problem is that, often, there is lack of consideration as to whether AI is capable of doing the job it was selected to perform.

Furthermore, vulnerabilities with AI continue to persist. Hurlburt [69] noted that AI should be trusted only to a certain extent. However, the consideration and act of trust toward selecting AI to complete tasks is better suited for the individual evaluating the AI tool than the task itself.

We also advocate for some level of skepticism regarding the decisions made by AI. The amount of skepticism necessary for the most accurate clinical decisions depends on the capability of the clinician and AI system. Suppose the benefits and constraints of an AI tool are understood. In that case, decision-making about whether to use the tool entails placing trust (binary in nature), rather than considering the extent to which a tool with predefined specifications can be trusted. The authors’ effort to analogize trust in AI with trust in humans is remarkable in the literature on AI involving trust. For example, in the study by Hengstler et al [70], trust in AI used in tools such as autonomous vehicles and medical assistance devices was investigated [70]. They sought to draw an analogy between applied AI in vehicles and human social interaction [70]. Their focus was on understanding the relationship between humans and automation, to understand how trust is built. They concluded that trust in AI is inextricably linked to the trust that individuals have in the firm that created the AI. The philosophical approach to this research is very different from that of researchers examining trust in technology in general [64,65]. The focus of researchers was on establishing that trust in technology and trust in humans are entirely different concepts. Therefore, the nature of AI as a form of intelligence designed to be similar to human intelligence can affect how AI is considered, even in scholarly research.

People’s trust in AI shares commonalities between trust formation in automation (non-AI technology) and interpersonal trust (trust in humans). Glikson and Woolley [71] discussed previous studies involving human trust in AI. They noted that there are critical differences between AI and other technologies, which impact how trust forms and works, similar to both human and technology trust. Cognitive and emotional trust in AI are related to both the representation of the AI, whether robotic, web-based, or embedded, and the level of intelligence of the AI system. These factors are integral to establishing people’s cognitive and emotional trust in AI. In the scope of AI use in health care, the conceptualization of AI as having some anthropomorphic qualities becomes increasingly visible. Kerasidou [72] examined the use of AI in health care, focusing on the issues of empathy, compassion, and trust. She noted that these are characteristics that people should not value in AI. However, AI in medical treatment plays a role where AI completes several tasks that humans traditionally complete. The technology must be task-oriented and support humans in health care by performing more activities related to the emotional and comfort aspects of patients’ treatment. Together, these findings contribute further support for AI, where the tool can fill a supportive role and enable humans to hold a position where trust would be beneficial to their health care delivery.

### Patient Safety and Informing Decisions With AI

The most fundamental aspect of medical care is the promise of physicians to not harm (patient safety). The Hippocratic Oath is the standard that health care professionals must follow when working with patients. Therefore, understanding how AI impacts patient safety is critical for this study.

Health care AI studies have positively contributed to drug development, personalized medicine, and patient care monitoring [73-76]. AI has also been incorporated into electronic health records to identify, assess, and mitigate threats to patient safety [77]. Recent studies and reviews have primarily focused on the performance of AI at the diagnostic level, such as disease identification [78-83], and AI robotics in surgery and disease management [84-87]. Other studies have also implemented AI technologies to assist at the clinical level, including for assessing fall risks [88] and medication errors [89,90]. However, many, if not all, of these studies have not implemented AI in a clinical setting or have been used by clinicians for routine clinical activities. Therefore, we noted a lack of evidence that can confirm the positive impact of AI on patient safety outcomes in real life.

The impact of AI on patient safety substantially depends on how clinicians correctly comprehend AI output (information and recommendation) and accordingly make clinical decisions. In other words, misinterpretation of the AI output may mislead clinicians and encourage them to make wrong clinical decisions—putting patient safety at risk. With the integration of AI, the role of technology shifts from merely *delivering information* to *information identification* and *decision-making*, therefore, enunciating the importance of clinician-AI interaction and collaborative decision-making. Most decision-making literature in the context of health care focuses on shared decision-making (clinician-patient) and its impact on patient safety. However, no studies have considered the significant role that AI can play in clinical decision-making (clinician-AI) and patient safety. Woolf et al [91] believed that an informed choice should occur in an interpersonal manner. Légaré et al [92] also discussed the importance of increasing the use of shared decision-making.

Nevertheless, the critical findings of health care decision-making literature may also apply to AI-based decisions. For instance, Edwards et al [93] found that in shared decision-making between the patient and clinician, the patient’s degree of health literacy determined their ability to understand their treatment (creating a shared mental model between the clinician and patient). Similarly, clinicians’ AI literacy will assess their ability to comprehend AI outcomes and make informed clinical decisions, thus ensuring treatment adherence and safety.

The importance of information interpretation and analysis, in general, has been well acknowledged in the literature [94]. For example, Tuffaha et al [95] discussed using the value of information analysis in health care as a model to support health care decision-making approaches. The value of the information analysis approach supports the measurement of decision uncertainty and assessment of the evidence’s sufficiency to support technological implementation. Bindels et al [94] supported the use of the value of information analysis in health care decision-making. Although the value of information analysis is a practical approach to decision-making, there is a lack of implementation of AI and studies analyzing its impact on clinical decision-making and patient safety. These findings provide evidence that the issue of safety must receive further focus in the form of empirical research to inform patient safety and informed decisions regarding AI. The current body of research includes a rich collection of studies focused on using AI in tasks and decision support roles where the potential exists that users or those dependent on AI use are at risk of possible harm from AI technology.

## The Proposed Framework

**Figure 2:**
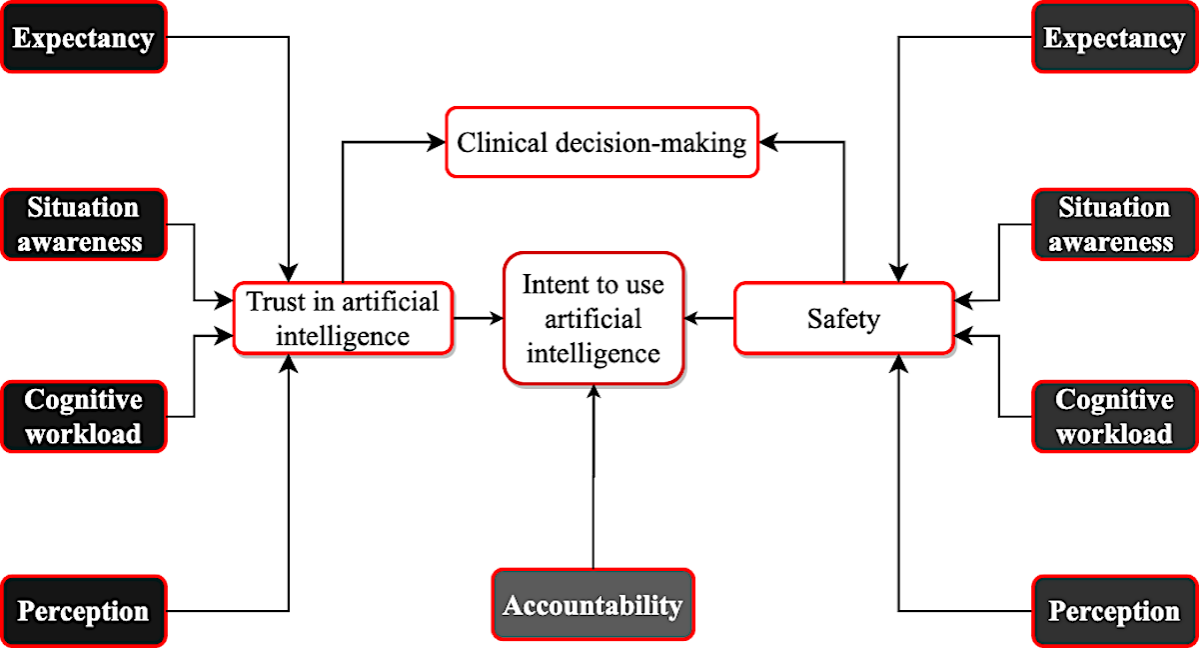
Ecological validation of artificial intelligence—trust, safety, and decision-making using artificial intelligence.

On the basis of the literature discussed previously, this study proposed the following conceptual framework (Figure 2). The framework emphasizes clinicians’ cognitive functions and perceptions regarding AI, concerning their trust in the technology along with perceptions of patient safety (risk). In addition, the framework emphasizes the cognitive functions of *situation awareness, workload, expectancy (performance and effort), trust, patient safety, clinicians’* *perceptions of AI, and perception of AI accountability*.

To explore this conceptual framework for describing clinician-AI interactions in clinical decision-making, each independent variable has operational precedent in the cognitive human factors and behavioral economics literature. There are numerous measures of situation awareness, including, but not limited to, the 3-level model of Endsley, perceptual cycle model [96], and activity theory model [97]. Similarly, *workload* has numerous and moderately competing operationalizations based on profession or occupation, including, but not limited to, scientific and clinical jobs and occupations [98,99]. Regarding *perceptions of AI*, there is relatively less precedent operationally [100,101]. Similar to the independent variables in the descriptive model of clinician-AI interactions, the dependent variables of *trust* *in AI* and *perceptions of patient safety* have numerous operationalizations across the computer science and health care literature [102,103].

Accordingly, the framework constitutes a sociocognitive approach that extends the theories of distributed cognition and, thus, accounts for the ecological validity of AI. The model leverages the measures that the studies reviewed in the previous sections imply (and often explicitly state), which must be included to understand the ecological validity of any model of human-AI interactions in decision-making. These validated and well-established scales include the modified National Aeronautics and Space Administration’ task load index [104], extended unified theory of acceptance and use of technology model [105], multi-item and previously validated scales for trust [106], and Mission Awareness Rating Scale [107] for situation awareness [108]. Inherently, cognitive workload and situation awareness are operationalizations of bounded rationality [109], and expectancy and perceptions are operationalizations of motivation and risk, respectively [110].

The real-life decision-making process deviates from the neoclassical or rational model of decision-making, which assumes perfect information and unlimited absorptive capacity, time, energy, and other resources—as implied in the framework. The underlying theory for the model is the expectancy-value theory of motivation, which posits that the probability of a specific decision to behave in a particular way (ie, AI-derived decision by a clinician) is dependent on the extent to which the decision maker believes that the specific behavior will elicit an intended outcome (ie, patient safety). The model can be illustrated differently based on the quantitative modeling of future researchers. The framework highlights the shaping factors that are likely to influence clinicians’ willingness to use an AI system. The framework captures the way in which the factors influence clinicians’ intention to use AI in their clinical workflow. In other words, future researchers can leverage this framework to explore the factors that influence clinicians’ cognitive function regarding the use of an AI system and, consecutively, impact the perception of patient safety or risk, trust in AI, and intent to use the technology. Subsequently, the framework also enables us to understand whether and how AI can influence clinical decision-making.

## Abbreviations

AI: artificial intelligence
HFE: human factors and ergonomics
HIT: health information technology
SEIPS: Safety Engineering Initiative for Patient Safety
TRL: technology readiness level
